# Assessment of Patient Satisfaction Regarding Clinic Visits in Riyadh, Kingdom of Saudi Arabia: A Cross-Sectional Study

**DOI:** 10.7759/cureus.65958

**Published:** 2024-08-01

**Authors:** Waqar Farooqi, Talal M Abukaram, Tarfah Alsulaiman, Arwa M Wadaan, Roha Sitwat, Haya AlDawood, Lamis M Gubran, Dana N Alsayed, Sumaya A AlHussain, Raghad Alhayyaf

**Affiliations:** 1 Internal Medicine, Almaarefa University, Riyadh, SAU; 2 College of Medicine, Almaarefa University, Riyadh, SAU; 3 College of Medicine, King Khalid University, Riyadh, SAU; 4 Biomedical Sciences, Aga Khan University Hospital, Karachi, PAK; 5 Medicine and Surgery, Almaarefa University, Riyadh, SAU

**Keywords:** visits, follow-ups, clinics, satisfaction, patient

## Abstract

Background

Understanding patient experiences and opinions is crucial to improving the quality of treatment given as healthcare services in Riyadh continue to expand. This study attempts to evaluate various aspects of patient satisfaction with clinic visits.

Objectives

To assess and analyze patient satisfaction with clinic visits in Riyadh, Kingdom of Saudi Arabia, in order to identify areas for improvement and enhance the overall quality of healthcare services in the region.

Methods

This cross-sectional study collected data from 350 adults aged 18 and above in Riyadh, Kingdom of Saudi Arabia. A paper-based questionnaire was distributed using a snowball convenience sampling technique at various locations. The survey assessed different aspects of patient satisfaction, including demographics, accessibility, quality of care, and patient experience. Ethical approval was obtained, and informed consent was acquired from all participants.

Results

The study's demographic distribution revealed that the majority of participants were female (77.4%), with the largest age group being 24-35 years old (34.9%). Saudi nationals constituted the majority (72.6%). Regarding accessibility and convenience of healthcare services, a significant proportion of participants agreed that the distance between their residence and the health center was reasonable (73.4%). However, opinions were mixed regarding waiting times, with some considering it reasonable (47.4%) and others disagreeing (25.4%). Participants also had varying views on appointment availability, with a notable percentage finding it difficult (33.7%). In terms of continuity of care and communication, most participants agreed that the clinic proactively contacts them for appointments (67.4%), but there were mixed responses regarding the ease of transferring patients to a hospital (37.7% agreed, 13.1% disagreed). The agreement on seeing the same doctor at each visit was moderate (41.1%), and a majority agreed that doctors had easy access to medical records (74.9%). Regarding the quality of care and patient experience, most participants agreed that doctors treated them with respect (83.7%) and that nurses and staff members were respectful and cooperative (54.3%). The majority agreed that health centers provided services during emergencies (78%). In terms of evaluating the quality of medical services and facilities, most participants agreed that vital signs were checked during each visit (78.6%), while satisfaction with laboratory facilities was moderate (60.3%). When it came to doctor-patient communication and counseling, most participants agreed that doctors provided detailed information about their disease and medications (73.4%) and addressed patients' queries (74.9%). However, some participants said that doctors did not inform them well about their disease (23.4%). Most participants agreed that doctors showed empathy and friendliness (73.7%) and allocated adequate time during visits (71.7%). However, satisfaction with post-visit accessibility to the doctor was mixed (35.1% agreed it was easy).

Conclusion

The findings revealed that while participants expressed satisfaction with certain aspects of care, there were areas requiring improvement. These areas included reducing waiting times, enhancing appointment availability, improving transfer procedures, ensuring consistency in doctor-patient relationships, and enhancing communication and counselling.

## Introduction

The assessment of healthcare service quality is significantly contingent upon the contentment and fulfillment of patients. Patient satisfaction serves as a pivotal metric in the evaluation and appraisal of the effectiveness, efficiency, and overall standard of care provided within healthcare settings. This critical measure not only reflects the extent to which patient needs and expectations are met but also underscores the importance of patient-centered care and the delivery of services that align with individual preferences and requirements [1-3]. It is associated with a positive patient-physician relationship, leading to improved adherence to treatment plans [3]. Patient satisfaction is influenced by various factors, including the quality of services provided and patients' perceptions of the environment, cleanliness, tranquillity, waiting times, and physician-patient communication [3,4]. Researchers have found that age, illness severity, gender, income, health status, medical insurance, marital status, and family size all play a role in determining patient satisfaction [5,6]. Other researchers suggest that patient satisfaction may be affected by the patient's past experiences and the views of others, including family and friends [7-9]. Nevertheless, ensuring the quality of care has become a significant challenge in meeting patient expectations and satisfaction [10-12]. As an integral component of Saudi Vision 2030, the healthcare system in the country is undergoing substantial transformation, with a focus on enhancing accessibility and effectiveness as the primary indicators of patient care quality [13]. Therefore, it is crucial to conduct a study to assess and analyze patient satisfaction with clinic visits in Riyadh, Kingdom of Saudi Arabia, in order to identify areas for improvement and enhance the overall quality of healthcare services in the region. The objective of this research is to evaluate patient satisfaction with clinic visits in Riyadh, Kingdom of Saudi Arabia, and to identify the factors that contribute to patient satisfaction. The research aims to answer the following research question: What are the key determinants of patient satisfaction with clinic visits in Riyadh? By conducting this study, we aim to contribute to the existing body of knowledge on patient satisfaction and provide insights that can inform healthcare providers and policymakers in improving the quality of healthcare services. The findings of this research will help identify areas for improvement and guide the development of strategies to enhance patient satisfaction and overall healthcare service quality in Riyadh.

## Materials and methods

Setting and sample

This cross-sectional study employed a questionnaire to assess the satisfaction levels of Saudi Arabia's population with their clinic visits. The research was specifically conducted in Riyadh, the capital city of Saudi Arabia. The survey was distributed in paper format to the general Saudi population, with physical copies made available at various locations such as clinics, community centers, and public gathering places. The intended sample size was determined through a sample size calculation aimed at achieving statistically significant results. We used a confidence level of 95% and a margin of error of 5% for this calculation. Based on these parameters and the estimated population size of adults in Riyadh, we calculated that a minimum sample size of 350 participants would be sufficient to ensure the reliability and validity of our findings. Participants were required to meet specific inclusion criteria to ensure the relevance of the data collected. The inclusion criteria were being a resident of Riyadh, Saudi Arabia, being aged 18 years or older, and willingness to take part in the study. There were no gender restrictions for eligibility. Individuals who did not meet these criteria, such as those living outside Riyadh or under the age of 18, were excluded from the study. To enhance the diversity and reach of the sample, the snowball convenience sampling technique was employed. Initial participants were asked to refer others to participate in the survey, thereby expanding the pool of respondents. This technique helped in capturing a wide range of participants from different backgrounds and areas within Riyadh.

Ethical consideration

Approval was granted by the Institutional Review Board (IRB) at Almaarefa University, Riyadh, Saudi Arabia (IRB log number: 23-078). Prior to participation, all individuals provided informed consent after receiving a concise explanation of the study's goals and the criteria for eligibility. Participants were informed of their complete right to withdraw from the study at any time without facing any obligations.

Instruments and validation

The initial part of the survey focused on gathering sociodemographic information such as gender, age, nationality, education, marital status, occupational status, and household income. The next section comprised a questionnaire that addressed different aspects related to the accessibility and convenience of healthcare services. The third section examined the continuity of care and communication between patients and healthcare providers. The fourth section evaluated the quality of care and the experiences of patients. The fifth section assessed the quality of medical services and facilities. Finally, the sixth section examined doctor-patient communication and satisfaction. Participants were given three response options: Agree, Disagree, or Not Sure. Before data entry, we made sure that the surveys were accurate and complete. The questionnaire underwent validation through both content and face validity.

Translation procedure

The questionnaire was translated into Arabic by two native speakers of the language. To ensure accuracy and clarity, a thorough review process was carried out to identify and resolve any inconsistencies, ambiguities, or possible misinterpretations that may have arisen from the independent translations. Any discrepancies or disagreements identified during the review were carefully examined and resolved through collaborative discussions involving the translators.

Statistical data analysis

The data that was gathered was analyzed using Statistical Package for the Social Sciences (IBM SPSS Statistics for Windows, IBM Corp., Version 25.0, Armonk, NY). Descriptive statistics were utilized to summarize the responses, while inferential statistics were employed to investigate the relationships between the variables of interest. The findings were presented in both tables and text format. A p-value of less than 0.05 was deemed statistically significant.

## Results

Table 1 presents the distribution of responses based on gender, age, nationality, education, marital status, occupational status, and income. Among the participants, 77.4% were identified as female, while 22.6% were male. The largest age group represented was 24-35, accounting for 34.9% of respondents. Saudi nationals constituted the majority at 72.6%, with non-Saudis comprising 27.4%. The majority of participants (84.6%) had attained a university education. In terms of marital status, 50.9% were single, while 49.1% were married. The occupational status of participants varied, with students (31.4%) and private sector employees (18.6%) being the most prominent groups. Regarding income, 55.4% reported earning more than 10,000, while 27.4% reported earning between 5000 and 10,000.

**Table 1:** Demographic information of the participants N represents the frequency count of participants, while % represents the percentage that frequency accounts for out of the total data.

Parameters		N	%
Gender	Male	79	22.6
	Female	271	77.4
Age	18-23	95	27.1
	24-35	122	34.9
	36-47	64	18.3
	48-57	53	15.1
	60+	16	4.6
Nationality	Saudi	254	72.6
	Non-Saudi	96	27.4
Education	Elementary	1	0.3
	Intermediate	3	0.9
	Secondary	48	13.7
	University	296	84.6
	No formal education	2	0.6
Martial status	Single	178	50.9
	Married	172	49.1
Occupational Status	Student	110	31.4
	Self-employed	13	3.7
	Private sector employee	65	18.6
	Government employee	74	21.1
	Other or at-home	88	25.1
Income	<5000	60	17.1
	5000-10000	96	27.4
	>10000	194	55.4

In terms of the accessibility and convenience of healthcare services, participant responses in Table 2 indicate that 73.4% agreed that the distance between their residence and the health center was reasonable. However, 9.4% disagreed, and 17.1% were unsure about this aspect. Regarding the working hours at the clinic, 55.1% agreed that they were suitable for all family members, while 16.9% disagreed, and 28% were unsure. Regarding the time spent in the waiting room, 47.4% agreed that it was reasonable, while 25.4% disagreed, and 27.1% were unsure. When it came to getting an appointment, 37.1% disagreed that it was difficult, while 33.7% agreed, and 29.1% were unsure.

**Table 2:** The table illustrates the collected responses pertaining to the accessibility and convenience of healthcare services. Parameter represents the choices that participants have been given (Agree, Disagree, Not sure); N represents the frequency count of participants, while % represents the percentage that frequency accounts for out of the total data.

	Parameters	N	%
Distance from my residence to the health center is reasonable.	Agree	257	73.4
	Disagree	33	9.4
	Not sure	60	17.1
	Total	350	100
Working hours at the clinic are suitable for all family members.	Agree	193	55.1
	Disagree	59	16.9
	Not sure	98	28
	Total	350	100
Time spent in the waiting room is reasonable.	Agree	166	47.4
	Disagree	89	25.4
	Not sure	95	27.1
	Total	350	100
I find it difficult to get an appointment.	Agree	118	33.7
	Disagree	130	37.1
	Not sure	102	29.1
	Total	350	100

Table 3 presents the continuity of care and communication with healthcare providers; findings showed that 67.4% of participants agreed that the clinic proactively contacts them to inform about appointments, while 13.1% disagreed, and 19.4% were unsure. In terms of transferring patients to a hospital, 37.7% agreed that the procedure was easy, while 13.1% disagreed, and 49.1% were unsure. Regarding the consistency of seeing the same doctor at each visit, 41.1% agreed, 24.3% disagreed, and 34.6% were unsure. When it comes to doctors having easy access to medical records, 74.9% agreed, 4% disagreed, and 21.1% were unsure.

**Table 3:** The table presents the responses concerning the continuity of care and communication with healthcare providers. Parameter represents the choices that participants have been given (Agree, Disagree, Not sure); N represents the frequency count of participants, while % represents the percentage that frequency accounts for out of the total data.

	Parameter	N	%
The clinic contacts me to inform about the appointment.	Agree	236	67.4
	Disagree	46	13.1
	Not sure	68	19.4
	Total	350	100
In case a patient needs to be transferred to a hospital, the procedure is easy.	Agree	132	37.7
	Disagree	46	13.1
	Not sure	172	49.1
	Total	350	100
I see the same doctor at each visit.	Agree	144	41.1
	Disagree	85	24.3
	Not sure	121	34.6
	Total	350	100
Doctor has easy access to my medical records.	Agree	262	74.9
	Disagree	14	4
	Not sure	74	21.1
	Total	350	100

Table 4 presents the relation between the quality of care and patient experience; participant responses indicated that 83.7% agreed that doctors at the clinic treated them with respect, while 2% disagreed, and 14.3% were unsure. Regarding the behavior of nurses and other staff members, 54.3% agreed that they were respectful and cooperative, while 9.1% disagreed, and 36.6% were unsure. In terms of health centers providing services during emergency situations, 78% agreed, 3.4% disagreed, and 18.6% were unsure.

**Table 4:** The table depicts the collected responses concerning the quality of care and patient experience. Parameter represents the choices that participants have been given (Agree, Disagree, Not sure); N represents the frequency count of participants, while % represents the percentage that frequency accounts for out of the total data.

	Parameter	N	%
Doctors at the clinic treat me with respect.	Agree	293	83.7
	Disagree	7	2
	Not sure	50	14.3
	Total	350	100
Nurses and other staff are respecting and cooperative.	Agree	190	54.3
	Disagree	32	9.1
	Not sure	128	36.6
	Total	350	100
Health center provides health services in emergency situations also.	Agree	273	78
	Disagree	12	3.4
	Not sure	65	18.6
	Total	350	100

Table 5 presents the feedback from participants regarding the evaluation of the quality of medical services and facilities indicating that 78.6% agreed that vital signs were checked during each visit, while 9.1% disagreed, and 12.3% were unsure. In terms of satisfaction with the laboratory facilities at the clinic, 60.3% agreed they were satisfactory, 9.1% disagreed, and 30.6% were unsure. When it came to doctors thoroughly examining patients as needed, 61.4% agreed, 12% disagreed, and 26.6% were unsure. Lastly, 48% of participants agreed that the clinic's medical staff kept up to date with the latest medical breakthroughs, while 8.9% disagreed, and 43.1% were unsure.

**Table 5:** The table presents the gathered responses regarding the assessment of medical services and facilities quality. Parameter represents the choices that participants have been given (Agree, Disagree, Not sure); N represents the frequency count of participants, while % represents the percentage that frequency accounts for out of the total data.

	Parameter	N	%
Vital signs like blood pressure, temperature, pulse and weight are checked at each visit.	Agree	275	78.6
	Disagree	32	9.1
	Not sure	43	12.3
	Total	350	100
Laboratory facilities at the clinic are satisfactory.	Agree	211	60.3
	Disagree	32	9.1
	Not sure	107	30.6
	Total	350	100
The doctor examines me completely and thoroughly as needed.	Agree	215	61.4
	Disagree	42	12
	Not sure	93	26.6
	Total	350	100
The clinic's medical staff is up to date on the latest medical breakthroughs.	Agree	168	48
	Disagree	31	8.9
	Not sure	151	43.1
	Total	350	100

In terms of doctor-patient communication and counseling (Table 6), 73.4% of participants agreed that doctors provided detailed information about their disease and medications, while 7.4% disagreed, and 19.1% were unsure. When it came to addressing patients' queries, 74.9% agreed that doctors were satisfying, while 5.4% disagreed, and 19.7% were unsure. As for doctors making patients feel uninformed, 23.4% agreed, 54.3% disagreed, and 22.3% were unsure. Regarding the display of empathy and friendliness by doctors, 73.7% agreed, 6.6% disagreed, and 19% were unsure. In terms of the time allocated by the doctor, 71.7% agreed that it was adequate, 8.6% disagreed, and 19.7% were unsure. When it came to post-visit accessibility to the doctor, 35.1% agreed it was easy, 34.6% disagreed, and 30.3% were unsure.

**Table 6:** The table shows the responses about doctor-patient communication and satisfaction. Parameter represents the choices that participants have been given (Agree, Disagree, Not sure); N represents the frequency count of participants, while % represents the percentage that frequency accounts for out of the total data.

	Parameter	N	%
The doctor counsels me about my disease and medicines in detail.	Agree	257	73.4
	Disagree	26	7.4
	Not sure	67	19.1
	Total	350	100
The doctor satisfies me whenever I have any queries.	Agree	262	74.9
	Disagree	19	5.4
	Not sure	69	19.7
	Total	350	100
The doctor sometimes makes me feel like I'm naïve.	Agree	82	23.4
	Disagree	190	54.3
	Not sure	78	22.3
	Total	350	100
Overall, the doctors are friendly and show empathy.	Agree	258	73.7
	Disagree	23	6.6
	Not sure	69	19.7
	Total	350	100
Time given to me by the doctor is adequate.	Agree	251	71.7
	Disagree	30	8.6
	Not sure	69	19.7
	Total	350	100
It is easy to contact my doctor after the visit, in case I need it.	Agree	123	35.1
	Disagree	121	34.6
	Not sure	106	30.3
	Total	350	100

The cross-tabulation results in Table 7 highlight the relationship between age and various aspects of patient satisfaction. For healthcare service accessibility and convenience, the p-value was 0.065, indicating no statistical significance, with the highest satisfaction in the 60+ age group (37.5%) and the most dissatisfaction in the 24-35 age group (80.3%). Regarding continuity of care and communication, the p-value was 0.459, also not statistically significant, showing the highest satisfaction in the 60+ age group (56.3%) and the most dissatisfaction in the 36-47 age group (64.1%). In terms of quality of care and patient experience, the p-value was 0.109, not statistically significant, with the highest satisfaction in the 18-23 age group (87.4%) and the most dissatisfaction in the 24-35 age group (25.4%). For the assessment of medical services and facilities quality, the p-value was 0.476, not statistically significant, with the highest satisfaction in the 18-23 age group (60%) and the most dissatisfaction in the 36-47 age group (53.1%). Lastly, for doctor-patient communication and satisfaction, the p-value was 0.226, not statistically significant, with the highest satisfaction in the 48-57 age group (58.5%) and the most dissatisfaction in the 36-47 age group (57.8%). These p-values indicate that there are no statistically significant differences across different age groups for each aspect of patient satisfaction.

**Table 7:** The table illustrates the relationship between age and the data presented in all the tables. N (%) frequency is displayed outside the brackets, while the percentage is shown within the brackets.

Parameters	Age	Satisfied	Not Satisfied	Total	P-value
Cross-tabulation of healthcare service accessibility and convenience by age.	18-23	30 (31.6)	65 (68.4)	95 (27.1)	0.065
	24-35	24 (19.7)	98 (80.3)	122 (34.9)	
	36-47	13 (20.3)	51 (79.7)	64 (18.3)	
	48-57	19 (35.8)	34 (64.2)	53 (15.1)	
	60+	6 (37.5)	10 (62.5)	16 (4.6)	
Cross-tabulation of the continuity of care and communication with healthcare providers by age.	18-23	46 (48.4)	49 (51.6)	95 (27.1)	0.459
	24-35	51 (41.8)	71 (58.2)	122 (34.9)	
	36-47	23 (35.9)	41 (64.1)	64 (18.3)	
	48-57	23 (43.4)	30 (56.6)	53 (15.1)	
	60+	9 (56.3)	7 (43.7)	16 (4.6)	
Cross-tabulation of the quality of care and patient experience by age.	18-23	83 (87.4)	12 (12.6)	95 (27.1)	0.109
	24-35	91 (74.6)	31 (25.4)	122 (34.9)	
	36-47	54 (84.4)	10 (15.6)	64 (18.3)	
	48-57	40 (75.5)	13 (24.5)	53 (15.1)	
	60+	14 (87.5)	2 (12.5)	16 (4.6)	
Cross-tabulation of the assessment of medical services and facilities quality by age.	18-23	57 (60)	38 (40)	95 (27.1)	0.476
	24-35	71 (58.2)	51 (41.8)	122 (34.9)	
	36-47	30 (46.9)	34 (53.1)	64 (18.3)	
	48-57	27 (50.9)	26 (49.1)	53 (15.1)	
	60+	9 (56.3)	7 (43.7)	16 (4.6)	
Cross-tabulation of doctor-patient communication and satisfaction by age.	18-23	57 (60)	38 (40)	95 (27.1)	0.226
	24-35	63 (51.6)	59 (48.4)	122 (34.9)	
	36-47	27 (42.2)	37 (57.8)	64 (18.3)	
	48-57	31 (58.5)	22 (41.5)	53 (15.1)	
	60+	8 (50)	8 (50)	16 (4.6)	

## Discussion

The assessment of patient satisfaction regarding clinic visits in Riyadh, Kingdom of Saudi Arabia, is vital in understanding the quality of healthcare services provided and identifying areas for improvement. This study aimed to evaluate various aspects of patient satisfaction to enhance the overall quality of healthcare services in the region. The findings of this study provide valuable insights that can inform healthcare providers and policymakers in their efforts to improve patient satisfaction. The study included 350 participants above the age of 18, with a majority of female respondents (77.4%). The largest age group represented was 24-35 years old (34.9%). The majority of participants were Saudi nationals (72.6%), while non-Saudis constituted 27.4% of the sample. Regarding accessibility and convenience of healthcare services, the majority of participants agreed that the distance between their residence and the health center was reasonable (73.4%). This indicates that most patients find it convenient to access healthcare services in Riyadh. When it comes to the working hours of the clinic, more than half of the participants (55.1%) agreed that the working hours were suitable for all family members. This suggests that the clinics have taken into consideration the convenience of patients and their families when scheduling appointments. The study also examined the time spent in the waiting room. While 47.4% of participants considered it reasonable, 25.4% disagreed. This indicates that there is room for improvement in reducing waiting times and enhancing the overall patient experience in the waiting area. In terms of appointment availability, 37.1% of participants disagreed that it was difficult to get an appointment, while 33.7% thought that it was difficult. This suggests that there may be some challenges in accessing timely appointments, which should be addressed to improve patient satisfaction.

It is important to note that these findings are consistent with a previous study by Al-Sakkak et al. conducted in 2008 [14], which indicates that patients' satisfaction with the accessibility and convenience of healthcare services in Riyadh has remained relatively consistent over time. Continuity of care and communication with healthcare providers are crucial aspects of patient satisfaction. The study found that 67.4% of participants agreed that the clinic proactively contacts them to inform about appointments, indicating a positive communication practice. However, when it comes to transferring patients to a hospital, only 37.7% agreed that the procedure was easy, while 13.1% disagreed. This highlights the need for streamlining the process of transferring patients and ensuring seamless continuity of care [15]. Participants' opinions regarding seeing the same doctor at each visit were mixed, with 41.1% agreeing and 24.3% disagreeing. This suggests that efforts should be made to enhance consistency in doctor-patient relationships, as continuity of care plays a significant role in patient satisfaction [1]. The study also assessed the quality of care and patient experience. The majority of participants agreed that doctors at the clinic treated them with respect (83.7%), indicating a positive patient-physician relationship. Regarding the behavior of nurses and other staff members, 54.3% agreed that they were respectful and cooperative, highlighting the need for maintaining a supportive and respectful healthcare environment. Participants' feedback on the evaluation of medical services and facilities indicated that 78.6% agreed that vital signs were checked during each visit, indicating a satisfactory level of care. However, only 60.3% agreed that the laboratory facilities at the clinic were satisfactory, suggesting that improvements could be made in this area. In terms of doctor-patient communication and counseling, the majority of participants (73.4%) agreed that doctors provided detailed information about their diseases and medications. This highlights the importance of effective communication in ensuring patient understanding and engagement in their healthcare journey.

## Conclusions

This study provides valuable insights into patient satisfaction with clinic visits in Riyadh, Saudi Arabia, identifying both strengths and areas for improvement. The findings show that while patients are generally satisfied with the accessibility and quality of healthcare services, there are significant areas needing enhancement, such as reducing waiting times, improving appointment availability, streamlining transfer procedures, ensuring consistency in doctor-patient relationships, and enhancing communication and counseling. By addressing these areas, healthcare providers and policymakers can better meet patient expectations, leading to improved overall healthcare quality and patient satisfaction in Riyadh. These efforts are crucial for the continued advancement of patient-centered care in alignment with Saudi Vision 2030.
